# Ventricular bigeminy associated with myocardial ischemia in a dog with a colonic torsion: a case report

**DOI:** 10.1186/s12917-024-04001-2

**Published:** 2024-04-26

**Authors:** Charlotte Burns, Michele Barletta

**Affiliations:** Department of Large Animal Medicine and Surgery, College of Veterinary Medicine, University of Georgia, Athens, GA 30605 USA

**Keywords:** Colonic torsion, Dog, Myocardial ischemia, Ventricular bigeminy

## Abstract

**Background:**

Ventricular bigeminy due to myocardial ischemia has been reported in humans as well as in canine patients with obstructive gastrointestinal diseases. This is the first case report of ventricular bigeminy in a dog with a colonic torsion that resolved after fluid resuscitation and restoration of myocardial perfusion.

**Case presentation:**

An 11-year-old, male neutered mixed breed dog presented with a one day history of vomiting, tenesmus, and lethargy. Physical examination identified an irregular heart rhythm and intermittent pulse deficits. A ventricular arrhythmia represented by ventricular premature complexes (VPCs) organized in bigeminy, was appreciated on a 3-lead electrocardiogram (ECG) with a single lead (II) view. Abdominal radiographs confirmed a colonic torsion. Prior to anesthetic induction, ventricular bigeminy was non responsive to fentanyl or lidocaine. The patient was anesthetized and intravascular volume deficit was identified by dampened plethysmographic wave amplitude (plethysomographic variability), audible softening of the Doppler sound, and more pronounced pulse deficits. Fluid resuscitation was achieved with a combination of intravenous crystalloid and colloid fluid therapy comprising 7.2% hypertonic saline and 6% hetastarch. The patient’s cardiac rhythm converted to normal sinus after fluid resuscitation. The colonic torsion was surgically corrected. The patient recovered well from anesthesia and was ultimately discharged from the hospital 5 days later.

**Conclusions:**

The present case report highlights that myocardial ischemia can lead to ventricular arrythmias, such as ventricular bigeminy. This is the first documented case of ventricular bigeminy in the canine patient with a colonic torsion. Assessment of patient volume status and appropriate fluid resuscitation along with continuous electrocardiogram (ECG) monitoring are vital to patient stability under general anesthesia.

## Background

Ventricular bigeminy is an uncommon arrhythmia noted in both human and canine species. Ventricular extrasystoles have been shown to be markers for myocardial ischemia in humans [1] and have been reported in dogs with gastrointestinal obstructive surgical emergencies, such as gastric dilatation volvulus (GDV) as well as experimentally induced myocardial ischemia [2, 3]. To the authors’ knowledge this is the first case of ventricular bigeminy recorded in a canine patient with a colonic torsion due to possible transient myocardial ischemia.

## Case presentation

An 11-year-old 28 kg male neutered mixed breed dog was presented to the University of Georgia Veterinary Teaching Hospital for a one day history of vomiting, tenesmus, inappetence, and lethargy. The patient was hospitalized two months prior to the current event for post-operative care of a GDV performed at another facility as well as a right-sided pneumothorax caused by a possible diaphragmatic hernia developed intra-operatively. Prior to this incident, the patient had no other medical illness aside from osteoarthritis that was medically managed with carprofen (Rimadyl; Pfizer).

On physical examination, the patient was quiet, alert, and responsive with an elevated heart rate of 140 to 180 beats/min. An arrhythmia was suspected by an irregular heart rhythm on auscultation. No heart murmurs were appreciated. Femoral pulse quality was strong; however, intermittent pulse deficits were appreciated. Upon palpation, the abdomen was soft and the patient showed signs of minimal discomfort. Mucous membranes were pink and moist with a capillary refill time of less than 2 s. An elevated respiratory rate of 60 breaths/min was noted and all lung fields were clear at auscultation with no crackles or wheezes. Rectal temperature was 38.7 °C (101.7 °F). A 3-lead electrocardiogram (ECG) showed frequent ventricular premature complexes (VPCs) organized in bigeminy. Doppler blood pressure was 130 mmHg. An 18-gauge IV catheter was aseptically placed in the left cephalic vein and blood was collected for blood gas analysis, chemistry panel (NOVA), and complete blood count (CBC). Hematologic analyses showed a lactate of 2.5 mmol/L [0.9–4.2 mmol/L] along with a hematocrit of 30% [37.5–55.6%] and leukocytosis of 18,100 [4.2–13.2 × 10^3^ /μl], categorized by a mild neutrophilia of 13,300 [2.5–8.6 × 10^3^/μl]. A platelet count of 24,000 [136–429 × 10^3^ /μl] was also noted, but was suspected to be secondary to clumping. A manual count was performed which estimated 130,000 platelets. The remainder of the blood analysis was unremarkable aside from a mild hypochloremia metabolic alkalosis and mild ionized hypomagnesemia (Table 1). Abdominal radiographs were obtained which confirmed a colonic torsion with mildly decreased peritoneal serosal detail, mild diffuse splenomegaly, and incidental transitional vertebrae. An emergency exploratory laparotomy was elected.

**Table 1:** Preoperative Complete Blood Count (CBC) and blood gas/biochemistry values. A mild hypochloremic metabolic alkalosis and hypomagnesemia were noted. A manual platelet count estimated 130,000 platelets.

Blood Analysis	Patient Value	Reference Value
**Complete Blood Count (CBC)**		
Hematocrit	30.8	37.5–55.6%
White Blood Cell Count	18.1	4.2−13.2 × 10^3^/μL
Neutrophil Count	13.3	2.5−8.6 × 10^3^/μL
Platelet Count	24	136−429 × 10^3^/μL
**Blood Gas/NOVA**		
pH	7.44	7.29−7.43
pCO2	32.2	23.7−43.9 mmHg
pO2	57.7	48.1−235.3 mmHg
Sodium	145.2	143−151.1 mmol/L
Potassium	3.8	3.7− 4.8 mmol/L
Chloride	109.2	110.5−118.8 nmol/L
Ionized Calcium	1.1	1.1−1.4 mmol/L
Ionized Magnesium	0.40	0.43−0.58 mmol/L
Glucose	87	70−114 mg/dL
Lactate	2.5	0.9−4.2 mmol/L
BUN	14	9−27 mg/dL
Creatinine	1.1	0.7−1.5 mg/dL
Bicarbonate	22.3	14.5 −23.1 mmol/L

**Fig. 1:**
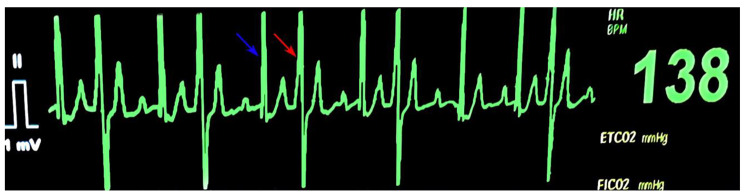
Lead II ECG recording from a multiparameter monitor. On the recording, ventricular premature complexes (VPCs) display a right bundle branch block morphology organized in bigeminy. The ventricular ectopic beats (red arrow) are premature, widened, and bizarre with no preceding p-wave. The ventricular ectopic beats (red arrow) alternate with sinus beats (blue arrows) in a 1:1 arrangement

During the pre-anesthetic period, maropitant (1 mg/kg, Cerenia; Zoetis) and 5 mL/kg/h lactated ringer’s solution (LRS) were administered IV. A Doppler blood pressure monitor was placed on the right forelimb over the palmar artery as well as a 3-lead ECG monitor with a single lead (II) view, which, as mentioned previously, showed ventricular premature complexes organized in bigeminy (Fig. 1). The heart rate fluctuated between 115 and 160 beats/min and, as the VPCs occurred, the Doppler became audibly softened. The femoral pulse deficits that were noted on exam also coincided with this arrhythmia and softening of the Doppler sound. The patient was premedicated with fentanyl (5 μg/kg, Fentanyl citrate; Pfizer) and lidocaine (2 mg/kg, Lidocaine hydrochloride; Hospira) IV. There was no improvement of the arrhythmia after the premedication drugs were administered, despite fentanyl having vagomimetic effects and lidocaine being a sodium channel blocker with anti-arrhythmic properties. General anesthesia was induced with ketamine (2 mg/kg, VetaKet; Akorn Animal Health) and propofol (4 mg/kg, PropoFlo 28; Zoetis) IV and the airway was secured with a 57 French cuffed Murphy endotracheal tube. Placement of the endotracheal tube was confirmed via direct visualization and presence of end-tidal carbon dioxide [P_E_’CO_2_]. The endotracheal tube was leak checked to hold at an airway pressure of 20 cmH_2_O and tied in place. General anesthesia was maintained on a rebreathing circuit with sevoflurane (Sevoflo; Zoetis) in 100% oxygen at 1 L/min [FiO_2_]. The concentration of inhalant was adjusted to maintain an appropriate anesthetic depth defined by ventromedial eye position, relaxed jaw tone, and lack of response to surgical stimulus. A 22-gauge catheter was placed aseptically in the left dorsal pedal artery for continuous direct blood pressure measurement. Direct systolic, diastolic, and mean arterial blood pressure (SAP, DAP, and MAP), respiratory rate [RR], heart rate [HR] and rhythm (by lead II ECG), pulse oximetry [SpO2], esophageal temperature [T,°F], P_E_’CO_2_ via side stream capnography, and end-tidal sevoflurane fraction [F_E_’_SEVO_] were monitored during the procedure with a multiparameter monitor (VetTRENDS V Plus Monitor; VetTRENDS). Constant rate infusions of fentanyl at 5–10 μg/kg/hr, lidocaine 50 μg/kg/min, and ketamine 1 mg/kg/hr IV were administered prior to the start of surgery.

After endotracheal intubation, intravascular fluid deficit was confirmed by performing a manual breathe hold at 20 cmH_2_O which produced a dampened plethysmographic wave amplitude (plethysomographic variability), audible softening of the Doppler sound, and more pronounced pulse deficits. At this time, a 10 mL/kg LRS IV fluid bolus was administered over 10–15 min. A point of care ultrasound of the thorax was performed during surgical preparation of the patient which showed subjectively underfilling of both the left and right ventricle. No free fluid within the pleural or pericardial space was appreciated. The patient was transported to the operating theatre and sterilely prepared for surgery, where he received additional boluses of 3 mL/kg of 7.2% hypertonic saline and 3 mL/kg of 6% hetastarch IV followed by a second 10 mL/kg LRS bolus IV, all administered over 10–15 min. After receiving additional fluid therapy, the patient’s heart rate decreased to 110–120 beats/min and the bigeminy (Fig. 1) resolved to a predominantly sinus rhythm with occasional/rare, single VPCs. The VPC related audible softening on the Doppler also resolved with improved palpable pulse quality. Mean arterial pressure decreased from 105 mmHg to 60 to 70 mmHg after fluid resuscitation, likely due to hemodynamic improvement of compensated shock, and maintained at this range for the rest of the anesthetic event. During manual ventilation, at peak inspiratory pressure of 20 cmH_2_O, there were no visual changes in amplitude of either the arterial blood pressure or plethysmographic waveform. The patient remained stable for the remainder of the surgery with a mild sinus tachycardia noted at the end of the procedure as the amount of inhalant anesthetic was reduced.

**Fig. 2:**
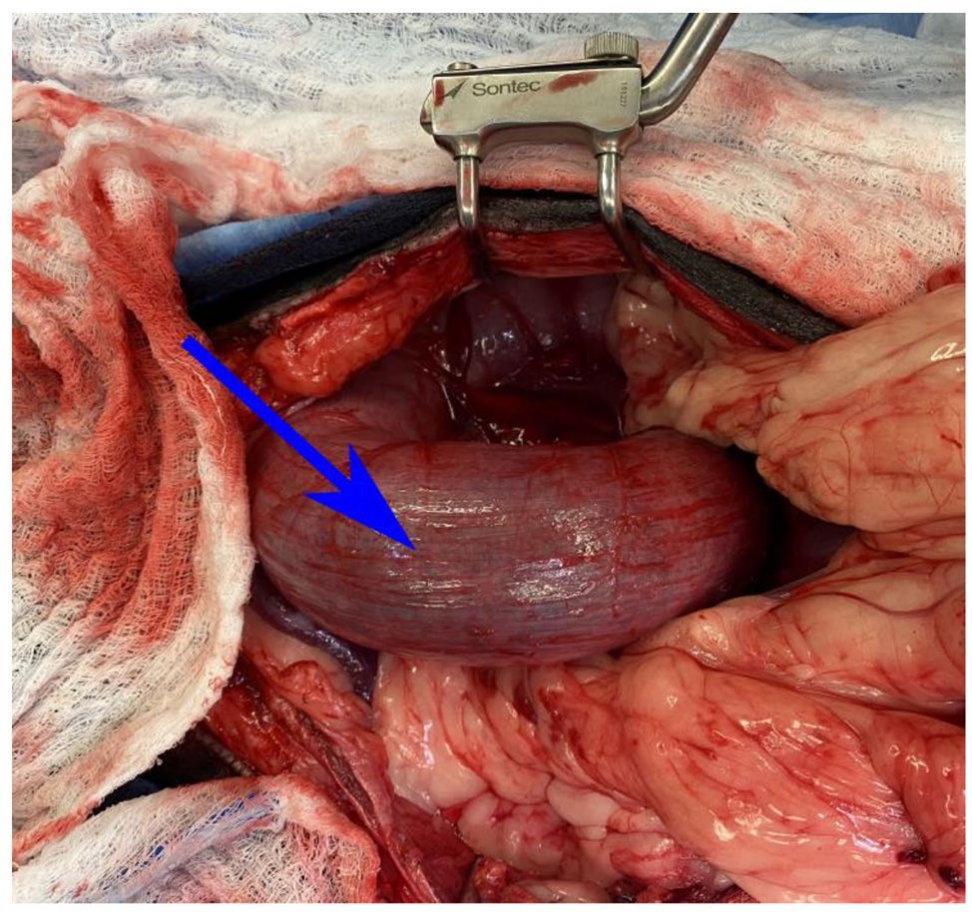
A colonic torsion was confirmed upon abdominal explore. The strangulated colon (blue arrow) is red and erythematous and rotate on its axis in a counter-clockwise fashion. The patient’s head is to the left of the image

A ventral midline celiotomy was performed which revealed a large gas distended colon (Fig. 2) that was de-rotated in a clockwise fashion improving its appearance. A 2.5 × 2 cm omental mass and an existing 1.5 cm gastropexy was identified on the right gastric body. A left sided colopexy was performed along with a gastrocolopexy and the previous right gastropexy was transected without complication.

The patient recovered without complications and was maintained on intravenous fluids, maropitant 1 mg/kg IV every 24 h, and methadone (0.1 mg/kg, Methadone hydrochloride; Mylan) IV every 6 h, along with continuous telemetry (Drager Infinity M540; Drager) for approximately 18 h to monitor for possible arrhythmias. The following morning the patient began receiving ondansetron (0.5 mg/kg, Zofran; Pfizer) IV every 8 h, and metoclopramide (2 mg/kg/day, Reglan; HIKMA) for nausea and diarrhea. Aside from an occasional sinus arrhythmia and atrial premature complexes (APCs), no further arrhythmias were noted postoperatively. A consultation with the cardiology service was performed and a grade 1/6 intermittent left apical systolic murmur was appreciated. An echocardiogram was performed by a cardiology resident overseen by a board certified cardiologist which showed trace mitral regurgitation and low normal systolic function, possibly due to the recent obstructive shock. No ectopy was appreciated during the echocardiogram. Histopathology of the omental mass indicated fat necrosis with fibrosis and lipogranulomatous steatitis. The patient was hospitalized for management of diarrhea for a total of 5 days prior to being discharged. The Holter monitoring and a follow-up visit were recommended; however, they were declined by the owner.

## Discussion and conclusions

While ventricular bigeminy has been reported in the dog, this is the first case of this arrhythmia in a patient with colonic torsion that was converted to sinus rhythm with intravenous fluid resuscitation. In a canine model investigating left stellectomy to prevent ventricular fibrillation caused by experimentally induced acute myocardial ischemia, ventricular bigeminy was noted after occlusion of the circumflex coronary artery [2]. Ventricular bigeminy has been reported in the dog after the administration of thiopental [4]. In a retrospective study of cardiac arrhythmias in dogs with gastric dilatation volvulus, ventricular bigeminy was noted as one of the ventricular arrythmias [3]. In contrast to this retrospective study which noted that periodic ventricular arrythmias began 12–36 h after surgery, the patient in this case report never developed ventricular arrhythmias post-operatively.

In the dog ventricular arrhythmias, such as VPCs, can be seen with primary cardiac disease such as dilated cardiomyopathy (DCM) or arrhythmogenic right ventricular cardiomyopathy (ARVC). However, VPCs can also be associated with a host of noncardiac diseases such as myocardial ischemia, electrolyte derangements, drug administration (barbiturates), or trauma. Idiopathic VPCs are also common and can be found in healthy individuals. VPCS can take several patterns such as bigeminy or trigeminy, where every second or third beat is a VPC, respectively [5]. One possible mechanism for the development of bigeminy is re-entry due to ischemia. Re-entry occurs when the electrical impulse splits into 2 separate impulses due to an ischemic injury. As one impulse is unable to be propagated due to the block, the second impulse will reach the blocked point from the opposite direction. If this occurs after the refractory period of the cardiac cells, a re-entry circuit develops, triggering premature complexes [6]. Ventricular bigeminy has been documented in people with underlying cardiomyopathies [7], or who had recently experienced acute myocardial infarcts [8], and in people with genetic diseases, such as catecholaminergic polymorphic ventricular tachycardia [9].

We suspect that the origin of the ventricular bigeminy in our patient was caused by hypovolemia leading to reduced diastolic filling, reduced cardiac output, decreased coronary perfusion, and acute myocardial ischemia. Hypovolemia was confirmed in this patient by noticeable plethysmographic variation during manual ventilation of the patient with a breathe hold of 20 cmH_2_O along with audible and palpable pulse deficits. In the human literature, both arterial blood pressure and plethysmographic waveform changes in mechanically ventilated patients have been shown to be sensitive indicators for hypovolemia [10–12]. Pulse pressure variation (PPV) is a dynamic preload-dependent variable that is derived from variations in the arterial pressure waveform due to changes in intrathoracic pressure during mechanical ventilation [13]. Plethysmography variability index (PVI) is a noninvasive preload-dependent variable obtained from the plethysomogrpahic waveform fluctuations of the pulse oximeter that can be used to identify fluid responsiveness [13, 14]. A study in healthy dogs anesthetized with sevoflurane and mechanically ventilated found that PVI can predict fluid responsiveness with moderate accuracy in comparison to PPV [13]. Restoration of intravascular volume likely improved coronary perfusion causing the return to normal sinus rhythm. The patient did not have significant underlying cardiac disease, which was confirmed by the echocardiogram performed post operatively and he did not receive arrhythmogenic drugs prior to the development of the ventricular bigeminy. A mild hypomagnesemia was noted on preoperative bloodwork, which is a risk factor for ventricular arrythmias. Hypomagnesemia can lead to shortening of action potential amplitude and duration as well as decreased resting membrane potential, ultimately predisposing to spontaneous automaticity [15]. However, based on the patient’s rapid response to fluid resuscitation, we suspected that hypovolemia, leading to compensated shock and subsequent myocardial ischemia, was the primary underlying cause of this arrhythmia.

Colonic torsion in the dog is a rare life threatening surgical emergency [16, 17]. Case series of colonic torsion and volvulus have been reported in young to middle-aged dogs of medium or large breeds [16–18]. Clinical signs comprised vomiting, lethargy, and abdominal pain [17, 19], which were consistent with the findings in our patient. Predisposing factors include history of gastrointestinal disease and previous gastropexy for treatment of GDV. In one retrospective series, 4 out of 6 dogs had previously developed a GDV and all had undergone a right side gastropexy prior to development of a colonic torsion/volvulus [17].

In conclusion, this case report highlights that acute myocardial ischemia can lead to ventricular arrhythmias that are responsive to fluid resuscitation and improved coronary perfusion. Patients diagnosed with colonic torsion should receive intravenous fluid therapy to maintain intravascular and coronary perfusion and electrical activity of the heart should be monitored before, during, and after general anesthesia.

## Data Availability

All material obtaining to this case report is available in this published article.

## Abbreviations

F_E_’ _SEVO_: End tidal sevoflurane fraction
FiO_2_: Inspired fraction of oxygen
HR: Heart rate
IM: Intramuscular
IV: Intravenously
Min: Minutes
P_E_’CO2: End tidal carbon dioxide
SAP; DAP; MAP: Systolic, diastolic, mean arterial pressures
T: Temperature
